# Ocean Acidification Refugia of the Florida Reef Tract

**DOI:** 10.1371/journal.pone.0041715

**Published:** 2012-07-27

**Authors:** Derek P. Manzello, Ian C. Enochs, Nelson Melo, Dwight K. Gledhill, Elizabeth M. Johns

**Affiliations:** 1 Cooperative Institute for Marine and Atmospheric Studies, Rosenstiel School of Marine and Atmospheric Science, University of Miami, Miami, Florida, United States of America; 2 Atlantic Oceanographic and Meteorological Laboratories, National Oceanic and Atmospheric Administration, Miami, Florida, United States of America; 3 Ocean Acidification Program, National Oceanic and Atmospheric Administration, Silver Spring, Maryland, United States of America; Swansea University, United Kingdom

## Abstract

Ocean acidification (OA) is expected to reduce the calcification rates of marine organisms, yet we have little understanding of how OA will manifest within dynamic, real-world systems. Natural CO_2_, alkalinity, and salinity gradients can significantly alter local carbonate chemistry, and thereby create a range of susceptibility for different ecosystems to OA. As such, there is a need to characterize this natural variability of seawater carbonate chemistry, especially within coastal ecosystems. Since 2009, carbonate chemistry data have been collected on the Florida Reef Tract (FRT). During periods of heightened productivity, there is a net uptake of total CO_2_ (TCO_2_) which increases aragonite saturation state (Ω_arag_) values on inshore patch reefs of the upper FRT. These waters can exhibit greater Ω_arag_ than what has been modeled for the tropical surface ocean during preindustrial times, with mean (± std. error) Ω_arag_-values in spring = 4.69 (±0.101). Conversely, Ω_arag_-values on offshore reefs generally represent oceanic carbonate chemistries consistent with present day tropical surface ocean conditions. This gradient is opposite from what has been reported for other reef environments. We hypothesize this pattern is caused by the photosynthetic uptake of TCO_2_ mainly by seagrasses and, to a lesser extent, macroalgae in the inshore waters of the FRT. These inshore reef habitats are therefore potential acidification refugia that are defined not only in a spatial sense, but also in time; coinciding with seasonal productivity dynamics. Coral reefs located within or immediately downstream of seagrass beds may find refuge from OA.

## Introduction

Ocean acidification (OA) is the global decline in seawater pH due to the uptake of carbon dioxide (CO_2_) by the surface ocean [1]. This uptake of CO_2_ reduces the concentration of carbonate ions [CO_3_^2−^] and leads to a decline in the carbonate mineral saturation state (Ω, where Ω = [CO_3_^2−^][Ca^2+^]/K′_sp_, and K′_sp_ is the apparent solubility product of a carbonate mineral). The saturation state of a given carbonate mineral is a key driver of inorganic carbonate mineral kinetics [2] and thought to be an important controlling factor on the biogenic calcification of that mineral (e.g., corals and their aragonite skeletons) [3]. Coral reef ecosystems are especially vulnerable as their continued persistence is dependent on the deposition of CaCO_3_ exoskeleton by scleractinian corals [4].

Despite these concerns, we still have only a rudimentary understanding of the spatial and temporal variability of carbonate chemistry within reef environments. There is a pressing need to ascertain which locations, habitats or regions may be relatively susceptible or even resilient to OA. This is a challenging undertaking which will take years, if not decades to unravel, as a given areas risk to OA will be a function of localized biogeochemical feedbacks that may locally alter the rates of OA [5], differing species-specific susceptibilities, and interactions with other stressors. The first step towards addressing this issue is to document the present day, baseline conditions of particular reef systems. Regions that are naturally high in CO_2_ and have low aragonite saturation states (Ω_arag_), such as upwelling areas in the eastern tropical Pacific, are OA “hotspots” in that rising CO_2_ will force the already low Ω_arag_ to potentially critical levels [6], [7]. However, the larger natural variability in Ω_arag_ found in upwelling areas could also make organisms there more tolerant to future OA [8]. Regardless, areas that act as natural CO_2_ sinks may serve as OA refugia because calcareous organisms will experience higher Ω_arag_ relative to the open ocean.

Seagrass beds are often located near coral reefs and the Florida Reef Tract (FRT) is no exception [9]. A non-continuous offshore barrier reef parallels the Florida Keys [10]. Between the Florida Keys islands and offshore barrier reef lies Hawk Channel that contains high abundances of seagrass habitat [9], [11]. Patch reefs are also found within Hawk Channel, and they often exist as isolated islands surrounded by seagrass beds [12]. Recent work has shown that calcification of calcareous macroalgae can be stimulated by CO_2_ uptake of seagrasses [13]. To determine if photosynthetic CO_2_ uptake associated with seagrass beds has the potential to create OA refugia, carbonate chemistry was repeatedly sampled across an inshore-to-offshore gradient in the upper, middle, and lower FRT over two years. During periods of heightened productivity, there is a net uptake of total CO_2_ (TCO_2_) which significantly increases aragonite saturation state (Ω_arag_) values on inshore patch reefs of the upper FRT.

## Materials and Methods

**Figure 1:**
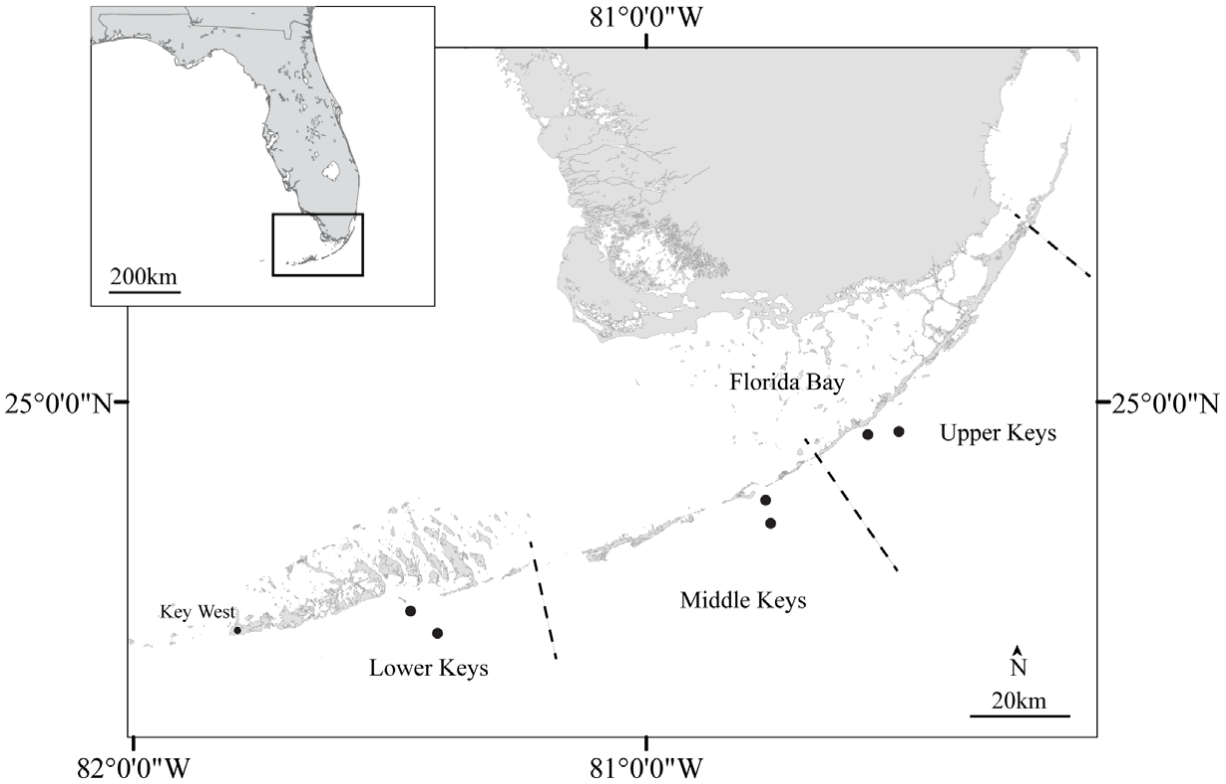
Map of Florida Keys portion of the Florida Reef Tract. Paired inshore and offshore sites where discrete seawater samples were obtained are indicated in the Upper, Middle, and Lower Keys. GPS coordinates are Upper Keys Inshore (24.93898N, 80.56272W), Offshore-24.9465N, 80.50207W); Middle Keys Inshore (24.81216N, 80.76075W), Offshore (24.76724N, 80.75227W); and Lower Keys Inshore (24.59723N, 81.45505W), Offshore (24.55141N, 81.40251W).

Discrete seawater sampling was started in April 2009 from paired inshore and offshore sites in the upper Keys (UK) (Fig. 1). No specific permits were required for the described field studies. Paired inshore and offshore sites were carefully selected so that they had the same depth of ∼4–5 m, as water depth is known to exert a strong influence on the impact that benthic biota have on seawater carbonate chemistry [7]. Samples were most often collected from the surface at 1 m depth. The timing and location of all discrete sampling is indicated in Table S1, whereas sample sizes are listed in Table 1. Additionally, seawater samples were taken from the surface (∼1 m depth) during repeat biophysical oceanographic cruises of the South Florida Program aboard the R/V F. G. Walton Smith (Table S1). Carbonate chemistry sample collection and analysis were performed as previously described [6], [7]. Briefly, total CO_2_ (TCO_2_) was measured coulometrically and total alkalinity (TA) was determined using gran titration. The remaining carbonate parameters were calculated from these values and *in situ* temperature with CO2SYS [14] using the dissociation constants of Mehrbach et al. [15] for carbonic acid as refit by Dickson and Millero [16] and Dickson [17] for boric acid.

**Table 1:** Mean values (± std. error of mean) for salinity, TCO_2_, nTCO_2_, TA, nTA, pCO_2_, and Ω_arag_ by season for Upper Keys (UK), Middle Keys (MK), and Lower Keys (LK) paired inshore and offshore sites.

Location	Season	N	N	Salinity (psu)	TCO_2_ (µmol kg^−1^)	nTCO_2_ (µmol kg^−1^)	TA (µEq kg^−1^)	nTA (µEq kg^−1^)	pCO_2_ (μatm)	Ω_arag_
**UK**										
**Inshore**	**Spring**	12	5	36.66 (0.141)	1868.0 (41.33)*	1784.1 (44.14)*	2298.5 (32.07)	2195.1 (37.12)*	257 (19.2)**	4.69 (0.101)***
	**Summer**	6	3	36.62 (0.226)	1925.5 (44.80)	1840.8 (46.43)	2309.2 (20.62)	2207.5 (24.46)	379 (35.2)	4.34 (0.253)
	**Autumn**	6	4	36.07 (0.310)	2085.3 (32.70)	2023.8 (38.53)	2386.4 (25.24)	2316.1 (36.94)	452 (38.5)	3.42 (0.157)
	**Winter**	4	3	35.82 (0.386)	2103.0 (84.40)	2054.0 (69.10)	2457.4 (72.22)	2400.7 (58.87)	299 (11.8)	3.91 (0.197)
**Offshore**	**Spring**	7	4	36.28 (0.062)	2036.7 (8.08)*	1964.8 (5.91)*	2386.1 (6.47)	2301.8 (5.41)*	377 (6.6)**	3.92 (0.067)***
	**Summer**	4	4	36.13 (0.157)	2027.0 (5.72)	1960.3 (13.97)	2381.3 (9.38)	2302.9 (15.37)	425 (18.2)	4.07 (0.148)
	**Autumn**	4	4	35.78 (0.324)	2053.0 (9.18)	2008.6 (16.69)	2374.8 (7.27)	2323.5 (16.61)	403 (13.3)	3.64 (0.069)
	**Winter**	4	4	36.22 (0.135)	2061.9 (19.78)	1992.6 (12.59)	2400.0 (21.09)	2319.3 (13.31)	340 (12.8)	3.73 (0.029)
**MK**										
**Inshore**	**Spring**	4	3	36.84 (0.071)*	1951.7 (15.03)*	1854.1 (12.91)*	2348.7 (12.08)	2231.4 (12.10)*	316 (8.7)*	4.39 (0.081)*
	**Summer**	4	4	37.29 (0.060)	1830.2 (46.18)*	1769.5 (85.47)	2223.3 (30.36)**	2123.6 (59.25)	338 (35.2)	4.32 (0.147)
	**Autumn**	4	3	35.93 (0.074)	2078.8 (43.36)	1956.2 (87.62)	2388.0 (35.01)	2277.2 (71.20)	404 (47.9)	3.47 (0.172)
	**Winter**	3	3	36.03 (0.448)	2112.5 (100.02)	2053.7 (112.63)	2479.1 (90.54)	2410.1 (108.99)	323 (23.4)	4.08 (0.150)
**Offshore**	**Spring**	3	3	36.23 (0.114)*	2030.3 (5.38)*	1961.4 (1.16)*	2346.9 (32.04)	2297.8 (7.49)*	384 (22.0)*	3.92 (0.055)*
	**Summer**	5	4	36.28 (0.329)	2014.7 (28.44)*	1944.6 (40.78)	2373.8 (22.32)**	2291.0 (40.12)	417 (19.5)	4.11 (0.123)
	**Autumn**	5	4	35.68 (0.447)	2023.4 (12.91)	1985.6 (19.54)	2367.5 (13.56)	2323.1 (18.96)	366 (9.62)	3.88 (0.093)
	**Winter**	3	3	36.24 (0.137)	2057.6 (22.51)	1987.3 (14.39)	2377.1 (12.45)	2296.0 (4.30)	366 (9.2)	3.54 (0.147)
**LK**										
**Inshore**	**Spring**	5	4	36.95 (0.291)	1839.2 (47.19)*	1743.2 (55.16)*	2216.7 (41.25)*	2100.8 (53.05)*	301 (28.1)	4.07 (0.097)
	**Summer**	5	4	36.75 (0.262)	1808.0 (70.04)	1720.9 (55.62)	2162.7 (50.59)	2059.2 (34.78)	367 (44.4)	3.90 (0.191)
	**Autumn**	5	3	36.22 (0.240)	2056.3 (29.07)	1987.7 (38.92)	2367.4 (28.71)	2288.2 (37.03)	395 (39.4)	3.47 (0.103)
	**Winter**	3	3	36.05 (0.196)	2103.8 (37.31)	2042.5 (26.67)	2455.8 (29.49)	2384.4 (16.09)	330 (30.0)	3.91 (0.130)
**Offshore**	**Spring**	5	4	36.33 (0.219)	1986.8 (20.54)*	1914.7 (30.76)*	2330.1 (21.53)*	2245.5 (33.77)*	375 (18.7)	3.83 (0.058)
	**Summer**	4	4	36.75 (0.533)	1949.0 (69.08)	1856.4 (61.34)	2324.6 (56.45)	2214.4 (49.48)	380 (36.4)	4.22 (0.137)
	**Autumn**	5	4	35.40 (0.478)	2023.7 (16.43)	2001.4 (15.06)	2360.9 (26.66)	2334.5 (12.46)	374 (13.0)	3.81 (0.165)
	**Winter**	3	3	36.28 (0.138)	2078.9 (32.30)	2005.5 (23.59)	2404.0 (27.36)	2319.2 (17.68)	359 (6.0)	3.60 (0.070)

Means are mean values for each sampling excursion by season. Significant differences between inshore and offshore as indicated with t-tests marked by symbol (*, p<0.05; **, p<0.01; ***, p<0.001). n, number of samples. N, number of trips.

Statistical comparisons were made between inshore and offshore sites in the UK, middle (MK), and lower (LK) Florida Reef Tract (FRT) after data were divided into seasons. Student's t-tests (α = 0.05 significance level) were used when data were normally distributed, whereas Mann-Whitney *U*-tests were used when they were not. One-way ANOVAs (or Kruskal-Wallis tests when data were not normal or homoscedastic) were used to ascertain significant effects of region (UK, MK, LK) and season within the inshore and offshore grouping. When significant differences were indicated, post-hoc t-tests were used to determine the relationship of regions/seasons.

Inshore values of TCO_2_, TA, pCO_2_, and Ω_arag_ were subtracted from their paired offshore values to determine the magnitude (Δ) of the gradient between inshore and offshore sites. A positive Δ indicates that inshore values are higher than offshore, whereas a negative Δ shows the opposite. TCO_2_ and TA values were normalized to salinity (nTCO_2_ = TCO_2_×35/S) prior to calculating Δ. Mean [± std. error of the mean (SE)] values by season, as well as the sum of the mean values across all seasons (Σ) are presented. Means represent the average gradient encountered by season, whereas the summed values indicate the annual net direction and magnitude of the gradient. TA-TCO_2_ plots were used to infer the dominant forcing mechanisms of the carbon cycle eliciting the inshore-to-offshore gradient in carbonate chemistry [18].

## Results

**Figure 2:**
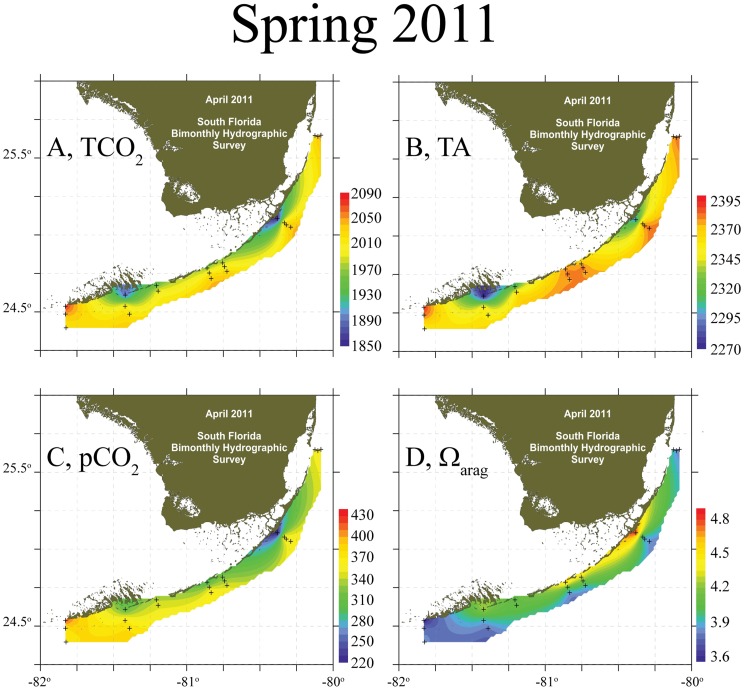
Carbonate Chemistry of Florida Reef Tract, Spring 2011. (A) Total CO_2_ (TCO_2_, µmol kg^−1^), (B) Total Alkalinity (TA, µEq kg^−1^), (C) partial pressure of CO_2_ (pCO_2_, μatm), and (D) aragonite saturation state (Ω_arag_) from April 2011 for the Florida Reef Tract.

**Figure 3:**
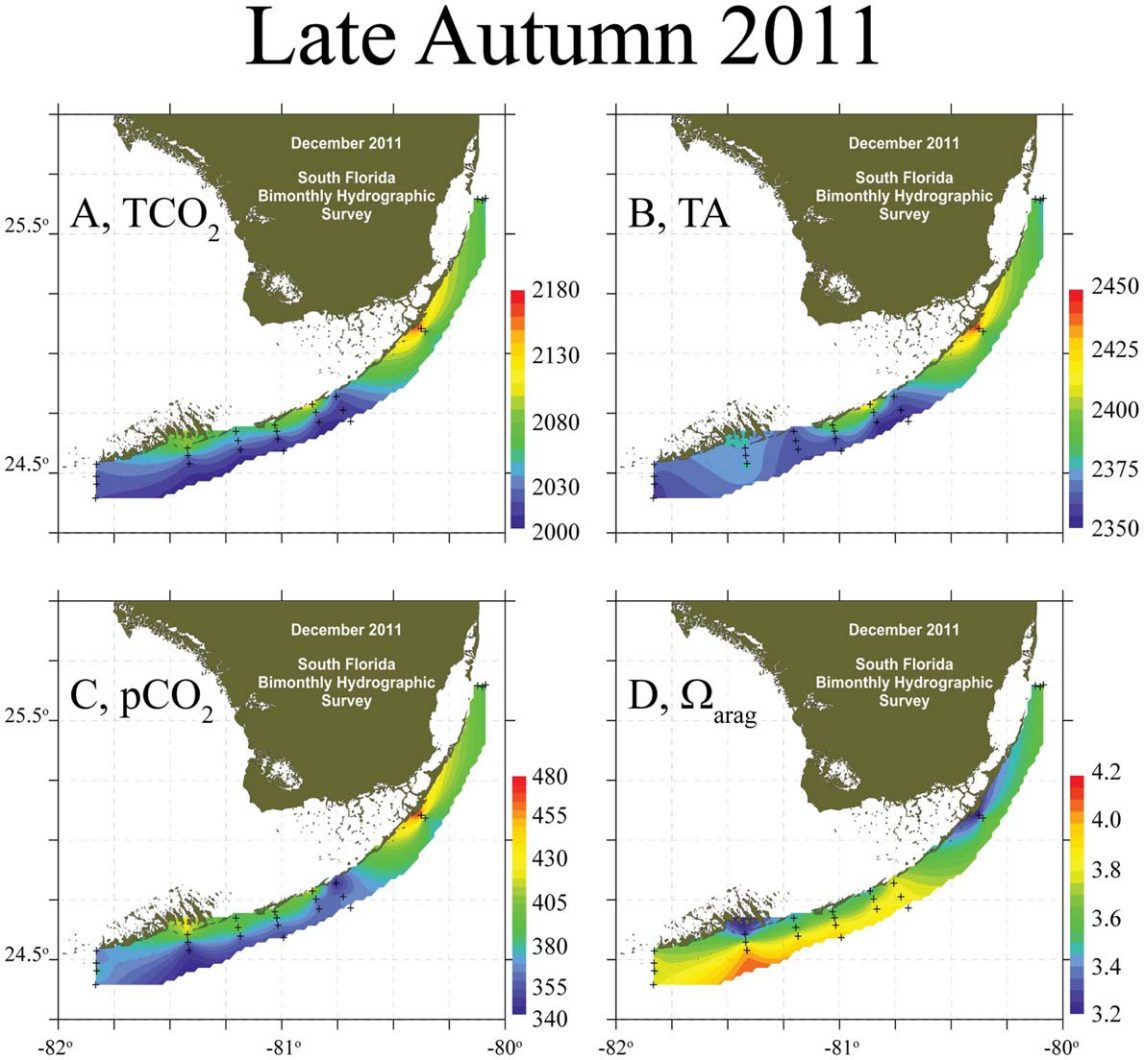
Carbonate Chemistry of Florida Reef Tract, Late Autumn 2011. (A) TCO_2_, (B) TA, (C) pCO_2_, and (D) Ω_arag_ from December 2011 for the Florida Reef Tract.

**Figure 4:**
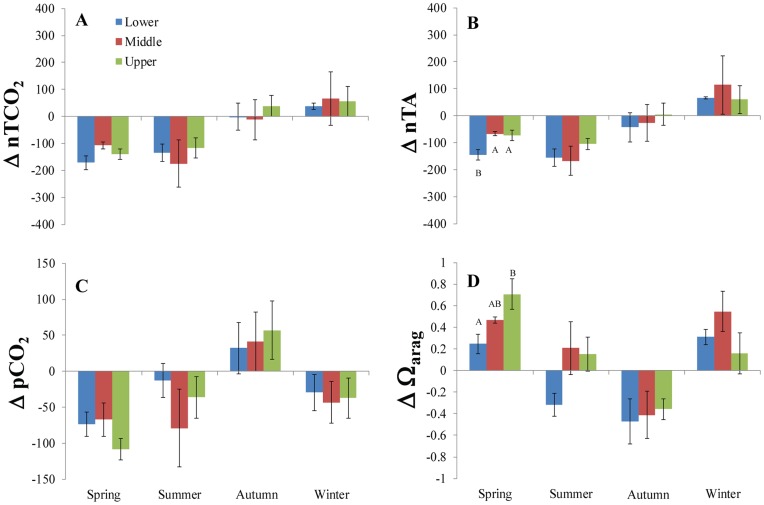
Seasonal Gradient in Carbonate Chemistry between Inshore and Offshore reef sites on the Florida Reef Tract. The difference (Δ) between inshore and offshore values of (A) nTCO_2_, (B) nTA, (C) pCO_2_, and (D) Ω_arag_ for the upper, middle, and lower Keys plotted by season. Values are means and error bars are standard error of the mean. Non-matching letters indicate significant differences (t-tests, p<0.05).

Inshore waters at all sites were depleted in both TCO_2_ and TA relative to offshore during spring and summer (Fig. 2) with the pattern generally reversing in autumn and winter (Fig. 3). In the spring, the significant inshore depletion of TCO_2_ was sufficient to elevate the Ω_arag_-values relative to offshore at all sites (Table 1; t-tests, p<0.05). The lack of significance between inshore and offshore pCO_2_ and Ω_arag_ measured in the LK during the spring was because of the large magnitude of TA decline inshore (Fig. 2B, 4; Table 1). The increase of inshore TCO_2_ in autumn depressed Ω_arag_ relative to offshore. By winter, the inshore TA increase compensated for increased TCO_2_ and caused an increase in Ω_arag_ relative to offshore once again (Fig. 4).

### Inshore-to-Offshore Gradient: Seasonal and Site Comparisons

**Figure 5:**
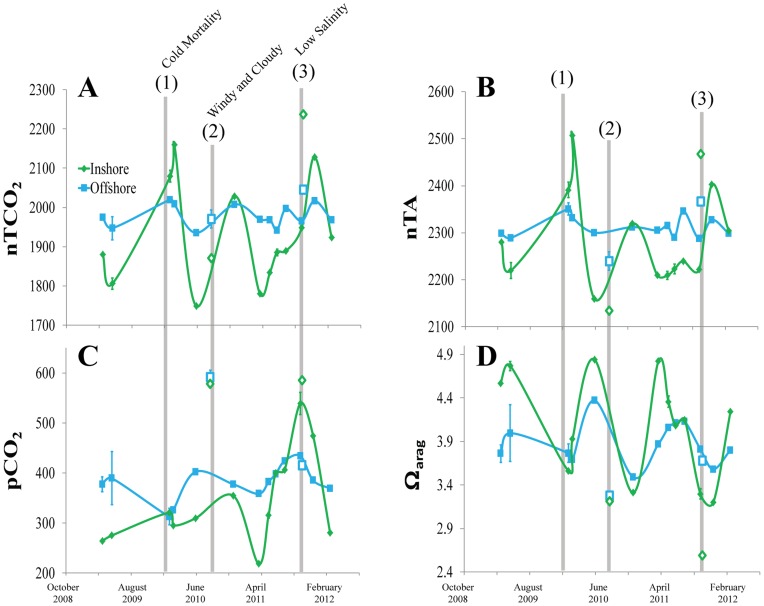
Time-series of Carbonate Chemistry at paired Inshore and Offshore reef sites in upper Florida Keys. Time-series of (A) nTCO_2_, (B) nTA, (C) pCO_2_, and (D) Ω_arag_ for paired inshore (green diamonds) and offshore (blue squares) reef sites in the Upper Florida Keys from April 2009 to February 2012. Anomalous events are noted for (1) sampling after cold-water mass mortality of inshore patch reefs in early 2010; elevated pCO_2_ and depressed Ω_arag_ associated with (2) high winds and overcast conditions in August 2010, and (3) low salinity in October 2011. Events (2) and (3) are represented by open diamonds (inshore) and squares (offshore) and are not linked to line as they deviate greatly from the seasonal pattern. The data points immediately after the cold-water mass mortality (1) are included because they represent important winter endpoints for 2009–2010.

The UK had the greatest magnitude of ΔΩ_arag_ (one-way ANOVAs, p<0.05, Fig. 4), due primarily to very high inshore Ω_arag_ values in spring (mean Ω_arag_ ± SE = 4.69±0.101, Table 1). The increase of inshore Ω_arag_ during the spring and summer is greater than its depression during autumn for both the UK and MK (Table 2), which is also evident in time-series data (Fig. 5). ΣΔpCO_2_ was negative for all sites (Table 2). A large negative ΣΔnTA value in the LK, which was greater than the large and negative ΣΔnTCO_2_ value, resulted in a negative ΣΔΩ_arag_. Also unlike the other sites, LK had a negative ΔΩ_arag_ during the summer (Fig. 4).

**Table 2:** Sum of differences between inshore and offshore sites across all seasons.

Site	ΣΔTCO_2_	ΣΔnTCO_2_	ΣΔTA	ΣΔnTA	ΣΔpCO_2_	ΣΔΩ_arag_
**Upper**	−144.4	−162.5	−71.0	−111.2	−125	0.66
**Middle**	−160.9	−228.0	−37.3	−147.3	−149	0.81
**Lower**	−244.9	−271.5	−243.3	−277.0	−84	−0.23

**Figure 6:**
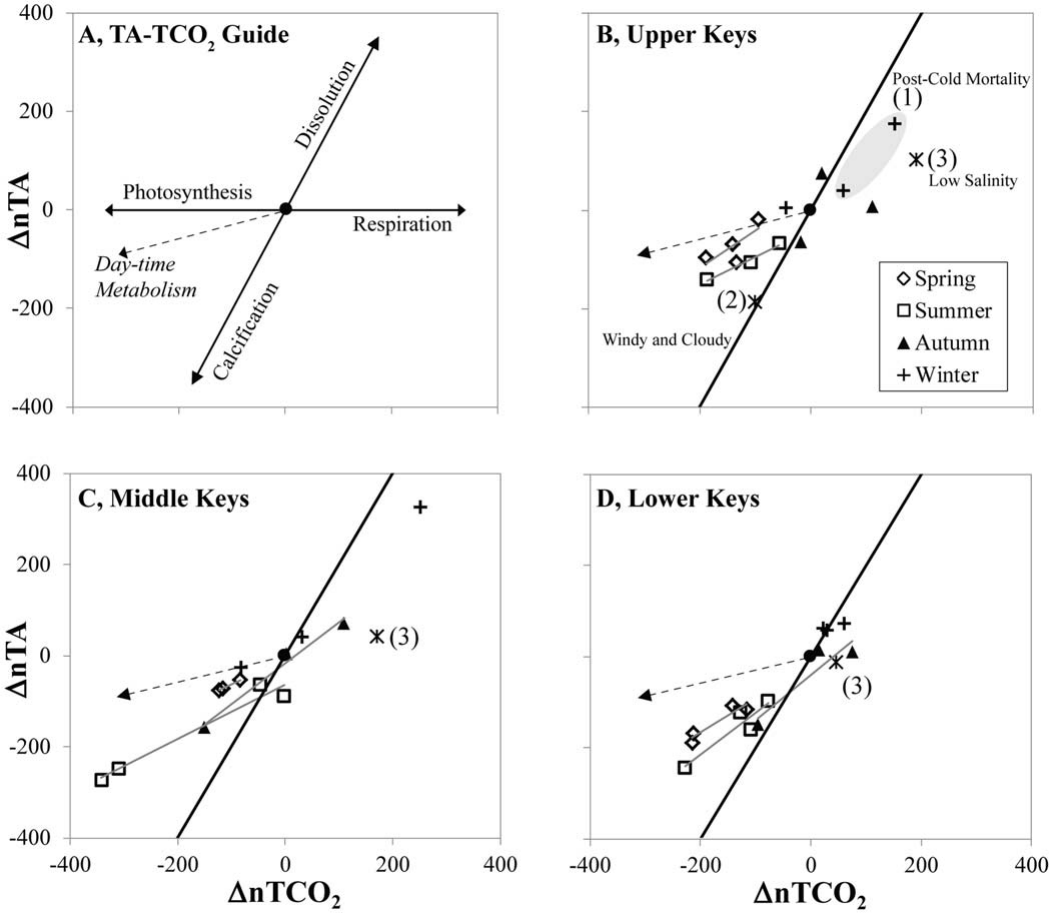
Total alkalinity (TA) vs. Total CO_2_ (TCO_2_) plots for upper, middle, and lower Florida Keys. (A) Guide to TA-TCO_2_ plots illustrating how net photosynthesis, respiration, calcification and dissolution affect location of points. TA and TCO_2_ are salinity normalized (S = 35) to allow comparison across seasons and sites. The reference offshore water mass is illustrated by the solid circle in the center of the plot. Vector addition of day-time metabolism assumes a photosynthesis/calcification molar ratio of 6 [17]. (B) Upper Keys, (C) Middle Keys, and (D) Lower Keys. The same three events listed in Fig. 5 are highlighted. (1) The samples obtained one and two months after the cold mortality are within the gray ellipse. The data point closer to the origin is from 16–17 February 2010, whereas the one farther removed is from 8 March 2010. Asterisks denote events (2) and (3).

The TA-TCO_2_ plots confirm the predominance of photosynthesis and calcification during the spring and summer at all sites (Fig. 6). The slopes of the UK and LK spring trendlines were no different (Fig. 6, Table S2). However, during the summer, the UK slope declined, indicating an increase in photosynthesis relative to calcification, whereas the LK slope increased, indicating an increase in calcification versus photosynthesis. The MK had large amplitude during summer because of the influence of Florida Bay waters that are depleted in TCO_2_ and TA during this time (Fig. 6C) [19]. The opposite occurred during the winter. During the winter months, net respiration and net dissolution were documented for all sites. In autumn, there was a scatter between the dominant spring/summer and winter processes for all sites.

### Paired inshore and offshore time-series

There was a large seasonal amplitude for both the inshore TCO_2_ and TA that was not apparent offshore (Fig. 5). Seasonally fluctuating temperatures result in corresponding seasonality in Ω_arag_ for both inshore and offshore waters, but the amplitude is enhanced for the inshore waters (Fig. 5). The inshore UK site had lower pCO_2_ most of the time except during or after anomalous events.

Three anomalous events impacted carbonate chemistry. First, there was an increase of inshore TCO_2_ and TA in the UK after the cold-water mass mortality of reef-building corals that occurred at inshore patch reefs in January 2010 (Fig. 5) [20]–[22]. This particular inshore site was seriously affected, as coral mortality in four species of corals was 83–100% (Manzello, unpub data). During August 2010, there were persistent winds at 5 to 7.5 m s^−1^ and overcast skies during sampling. As a result of lower than normal nTA, there was a spike in pCO_2_ and depression in Ω_arag_ at both sites, erasing any inshore-to-offshore gradient for these two parameters (Fig. 5). Interestingly, at this time the TA-TCO_2_ plot indicated net calcification in the absence of photosynthesis (Fig. 6B). Lastly, during October 2011, there was unseasonably high rainfall in south Florida and low salinity water was observed at all sites, with salinities inshore and offshore ranging from 32.04–32.685 and 34.035–34.835, respectively. At the inshore sites, low salinity was coincident with a spike in pCO_2_ and depression in Ω_arag_ (Fig. 5). All sites showed net respiration during this time (Fig. 6).

### Site and Seasonal comparisons

The carbonate chemistry was similar across the UK, MK, and LK when grouped by season and inshore/offshore (Figs. S1 and S2). A single exception was that UK inshore Ω_arag_ which, in addition to ΔΩ_arag_, was significantly higher than the LK in the spring (Fig. S1, t-test, p<0.01).

Seasonal effects were pronounced at the inshore sites, while the offshore sites were relatively stable (Table S3, Figs S1 and S2). The two exceptions were 1) pCO_2_ at the UK offshore site, which was highest in summer (t-tests, p<0.05), and 2) nTA at the LK offshore site, where there was a significant depletion in TA during summer (Fig. S2). The only other site where pCO_2_ varied significantly by season was the UK inshore site (Table S3). Ω_arag_ values were significantly higher in the spring versus autumn at all the inshore sites (p<0.05). Spring and summer TCO_2_ and TA values were significantly lower than the winter values at all inshore sites (p<0.05).

## Discussion

The inshore-to-offshore variability in carbonate chemistry on the FRT is primarily driven by large changes in TCO_2_, indicating that net primary productivity is the dominant mechanism forcing this gradient. The magnitude of the TCO_2_ depletion in the spring and summer is sufficiently large to elevate Ω_arag_-values despite a coincident decline in TA. The standing crop, abundance, and productivity of the dominant species of seagrass in the Florida Keys, *Thalassia testudinum*, follow a sinusoidal pattern, peaking from June to July, and reaching minimum values in January [11]. Macroalgae in the Florida Keys follow a similar seasonal pattern, whereby they increase in abundance from the spring to summer, then decline and reach a minimum in the winter [23], [24]. The temporal variation of these benthic primary producers coincide with the seasonal trend in TCO_2_ at the inshore sites.

Seagrass beds covered 46.2–55.6% of 3,141 km^2^ in the Florida Keys during a recent mapping study that included terrestrial and marine habitats [9]. The dominance of seagrass habitats within the Florida Keys lends support to the hypothesis that their productivity is responsible for the patterns in TCO_2_ reported herein. Seagrasses are usually net autotrophic ecosystems [25], which would explain the net uptake of TCO_2_ at all inshore sites (Table 2, Fig. 4). Net TCO_2_ uptake was also indicated by the persistence of the inshore-to-offshore gradient during nighttime sampling in May 2009, June 2010, April 2011, June 2011, and February 2012. Net autotrophy within seagrass meadows is due, in part, to low levels of herbivory, as <20% of seagrass productivity is consumed by herbivores [26]. Furthermore, seagrasses bury up to 50% of their production within their roots and rhizomes, which act as a CO_2_ sink [27], [28]. Reef areas with high macroalgal cover may also exhibit net CO_2_ uptake [29]–[31], though this carbon may be less permanently sequestered than in seagrasses [32]. A large fraction of the macroalgal production on reefs is directly respired, resulting in P/R ratios very close to 1, even for macroalgal dominated systems [33]. We hypothesize that the carbon sequestered by macroalgae is less persistent than that fixed by seagrasses. Macroalgae are either consumed by herbivores, or degraded microbially when the algae senesce on a seasonal basis. Either route represents a relatively short temporal fate whereby CO_2_ is ultimately respired back into the water column. There is no obvious route whereby the CO_2_ sequestered would be preserved like that of seagrass rhizomes and roots that are buried in sediments.

While reductions in CO_2_ associated with seagrass photosynthesis may ameliorate OA-related stress, several aspects of seagrass habitats are not conducive to coral growth and reef development. First, the soft substrate of seagrass beds is a poor habitat for both corals and other sessile reef-associated organisms [34]. While corals can survive as free-living motile colonies in areas of unstable substrate, these “coralliths” are usually small in size and have stunted growth rates [35]. Corals thrive on stable substrata, thus areas of antecedent hard bottom downstream or within seagrass areas may be important areas for coral persistence with accelerating OA.

Second, temperature fluctuations inshore on the FRT are high and potentially harmful to thermally sensitive coral species. Mean temperature values at our six sites from June 2010 to May 2011 were similar, ranging from 26.0 to 26.4°C. The variance in temperature was much greater at the inshore sites, as minimum and maximum temperatures were more extreme (Fig. S3). The greatest temperature fluctuations occurred in the MK, likely a result of the net outflow of Florida Bay waters through the wide channels in the middle Keys [36]. Florida Bay is shallow (average depth <3 m) and responds rapidly to changes in atmospheric temperatures [20]. At the inshore MK site, the minimum temperature was 14°C in Dec 2010 and the maximum temperature was >34°C in the summer of 2010 (Fig. S3). These temperatures are well outside the thermal tolerances of most coral species (18–30°C) [37]. Corals can tolerate conditions outside of this range if the duration and magnitude of temperature fluctuations is not too long or great, respectively. This 20°C range observed at the inshore MK site effectively restricts the proliferation of the majority of coral species. There are, however, a few especially hardy species, such as *Solenastrea bournoni*, and *Siderastrea radians*, but they are present in low abundances. Thus, while the ΣΔΩ**_arag_** was highest in the MK, thermal extremes preclude the viability of this area as a refuge for corals from OA. The range in temperature was less at the inshore sites in the UK and LK. These sites do experience extreme temperatures, but they are not as great in magnitude or as long in duration. This helps explain why isolated patch reefs are able to persist in these locations and not in the MK [38].

Overall, thermal conditions are more favorable at the offshore sites (Fig. S3). This is because the Florida Current effectively buffers these reef sites from the thermal extremes that occur close to shore [38]. Again, the MK offshore site, just like for the inshore sites, had the greatest range in temperatures (17.1°C), likely a result of the exchange of Florida Bay waters in the middle Keys [36]. This large temperature variability, by itself, is likely a primary factor of why the MK reefs are the most poorly developed of FRT [10]. The range in temperatures at the UK and LK offshore sites was less. However, it is important to realize that the FRT experiences some of the greatest temperature fluctuations documented for coral reefs. There are only a few locations, such as the Persian Gulf, where corals that form reef frameworks experience a wider range in temperatures (25°C) [39] . The FRT is at the upper and lower thermal limits for coral survival and reef development in the North Atlantic, due to cold water in the winter and warm water in the summer.

The FRT has experienced at least five warm-water bleaching events since 1987 that have encompassed the entire reef tract [40]. Inshore patch reefs experienced catastrophic coral mortality due to extreme cold weather in early 2010 that was an order of magnitude greater than any of the mortality associated with warm-water bleaching events to date [20]–[22]. Cold water stress, while rare, has repeatedly been identified as a primary limiting factor for the FRT and inshore sites are more prone to thermal extremes [38]. Although these sites may provide refuge from chronic OA stress, they will likely continue to be susceptible to acute cold weather events.

Intriguingly, the inshore patch reef environments of the upper FRT, which were found to have higher Ω_arag_ values than what has been modeled for the tropics prior to the industrial revolution [4], are known to have higher coral cover and faster coral growth rates compared to reefs offshore [41]. One hypothesis for the better condition of these nearshore patch reefs is that these areas may be more resistant to recurrent warm-water bleaching because of increased turbidity and chromophoric dissolved organic matter (CDOM) shading corals, partially reducing the photo-oxidative stress that occurs within the algal symbionts of corals during thermal stress [42], [43]. We suggest that the favorable Ω_arag_ conditions for these sites should also be considered as a contributing reason for their increased resilience.

Inshore patch reefs of the upper FRT may be OA refugia. The thermal extremes of the MK eliminate this area as a potential refuge. The ΣΔΩ_arag_ of the LK is negative, indicating that Ω_arag_-values inshore are more often lower than those offshore. Low TA values were measured during the summer for both the inshore and offshore LK sites (Figs. S1 and S2). Inshore TA values were sufficiently low to decrease Ω_arag_-values relative to those offshore (Table 1). It is not clear what is causing this apparent increase in calcification relative to photosynthesis. Tidally-driven flushing of the shallow waters between the numerous islands of the LK (see Fig. 1) may be resulting in the increased depletion of TA relative to TCO_2_, favoring CaCO_3_ precipitation that is not directly linked to photosynthesis. The bedrock of the LK islands is an oolitic deposit, whereas the Key Largo Limestone that makes up the UK and MK islands is coral reef derived [44]. Both facies were formed about 125,000 years ago, during the Pleistocence, when sea level was 4–6 m higher than the present day [45]. The same factors that favored the inorganic precipitation of CaCO_3_ (i.e., ooid shoals) in the LK, versus the biogenic coral reef deposits of the UK and MK, may be similar to what is occurring today on the FRT.

Seagrasses are also known to stimulate the dissolution of the calcareous sediments where their roots and rhizomes are buried [46]–[48]. This is because seagrasses pump a fraction of the oxygen produced during photosynthesis into their roots, which drives aerobic respiration and carbonate dissolution via CO_2_ production [46]. The addition of TA to seawater via this process has been suggested as a potential negative feedback to OA, as TA addition lowers pCO_2_ [47], [48]. Our data from the water column showed that TA often declined in concert with TCO_2_, indicative of net calcification (Fig. 6). Burdige and Zimmerman [46] showed that dissolution rate was a function of seagrass density and photosynthesitic productivity. Yet, TA was seasonally elevated at all sites (inshore and offshore) during the winter months (Table 1), and even more so at the inshore sites (Fig. S1, 4). This is when productivity of seagrasses on the FRT is at its minimum [11]. It is not clear if the TCO_2_ enrichment during autumn and winter is directly causing this elevated TA by driving dissolution. Whatever the mechanism, the elevation of TA during the winter offsets the TCO_2_ enrichment that occurs at this time, resulting in lower pCO_2_ and higher Ω_arag_ at the inshore sites (Fig. 4).

Seagrasses are carbon limited, whereas macroalgae are able to effectively utilize the abundant HCO_3_^−^ in seawater for photosynthesis [49]. Consequently, seagrass productivity is expected to be stimulated with OA [49]–[52], whereas macroalgae, already carbon-saturated, should not exhibit any increases in photosynthesis or growth [49]. For example, seagrasses exposed to high-CO_2_ conditions for 1 yr had increased reproduction, rhizome biomass, and vegetative growth of new shoots, which could represent a potential positive feedback to their ability to serve as OA refugia [51]. These results have implications for other calcifying organisms other than corals. Seagrasses, unlike shallow coral reefs, are an ecosystem that is not limited to the warm tropical latitudes. As such, the potential for seagrasses to locally buffer OA within coastal zones may be much greater than what is considered here and could even increase over time.

In summary, coral reefs in close proximity to seagrass beds may find refuge from OA. In the upper Florida Keys, inshore patch reefs have exhibited greater resilience relative to the offshore barrier reefs to a range of environmental stresses [41]. These inshore reefs are located where the uptake of TCO_2_ drives seasonal enhancement of Ω_arag_ (Fig. 4D). The natural sequestration of CO_2_ on the FRT may facilitate the high resilience of these sites.

## Supporting Information

Figure S1 **Carbonate Chemistry at Inshore Sites by Season.** Mean values for (A) nTCO_2_, (B) nTA, (C) pCO_2_, and (D) Ω_arag_ plotted by season for inshore sites from upper, middle, and lower keys. Error bars represent standard error of the mean. Means represent the average of mean values for each sampling excursion grouped by season. Non-matching letters indicate significant differences (t-tests, p<0.05).
(TIF)

Figure S2 **Carbonate Chemistry at Offshore Sites by Season.** Mean values for (A) nTCO_2_, (B) nTA, (C) pCO_2_, and (D) Ω_arag_ plotted by season for offshore sites from upper, middle, and lower keys. Error bars represent standard error of the mean. Means represent the average of mean values for each sampling excursion grouped by season.
(TIF)

Figure S3 **Seawater temperature at paired inshore and offshore sites.** *In situ* temperature data, collected every 30 min from paired inshore (green lines) and offshore (blue lines) sites for (A) upper, (B) middle, and (C) lower Keys. Depths of temperature values are 5 m.
(TIF)

Table S1 **Timing of discrete sampling at paired inshore and offshore sites in the upper (UK), middle (MK), and lower (LK) Florida Keys.** WS indicates sample taken aboard the R/V Walton Smith in coordination with the South Florida Program's repeat biophysical oceanographic cruises rather than small boat sampling (X).
(DOC)

Table S2 **nTA-nTCO_2_ trendline equations and R^2^ by site and season.**
(DOC)

Table S3 **Kruskal-Wallis results to identify seasonal differences within each of the six sites.**
(DOCX)
