# Supplementary figures and images for: Ocean Acidification Refugia of the Florida Reef Tract
Source: PLoS One. 2012 Jul 27;7(7):e41715. doi: 10.1371/journal.pone.0041715 (PMC3407208; doi:10.1371/journal.pone.0041715)

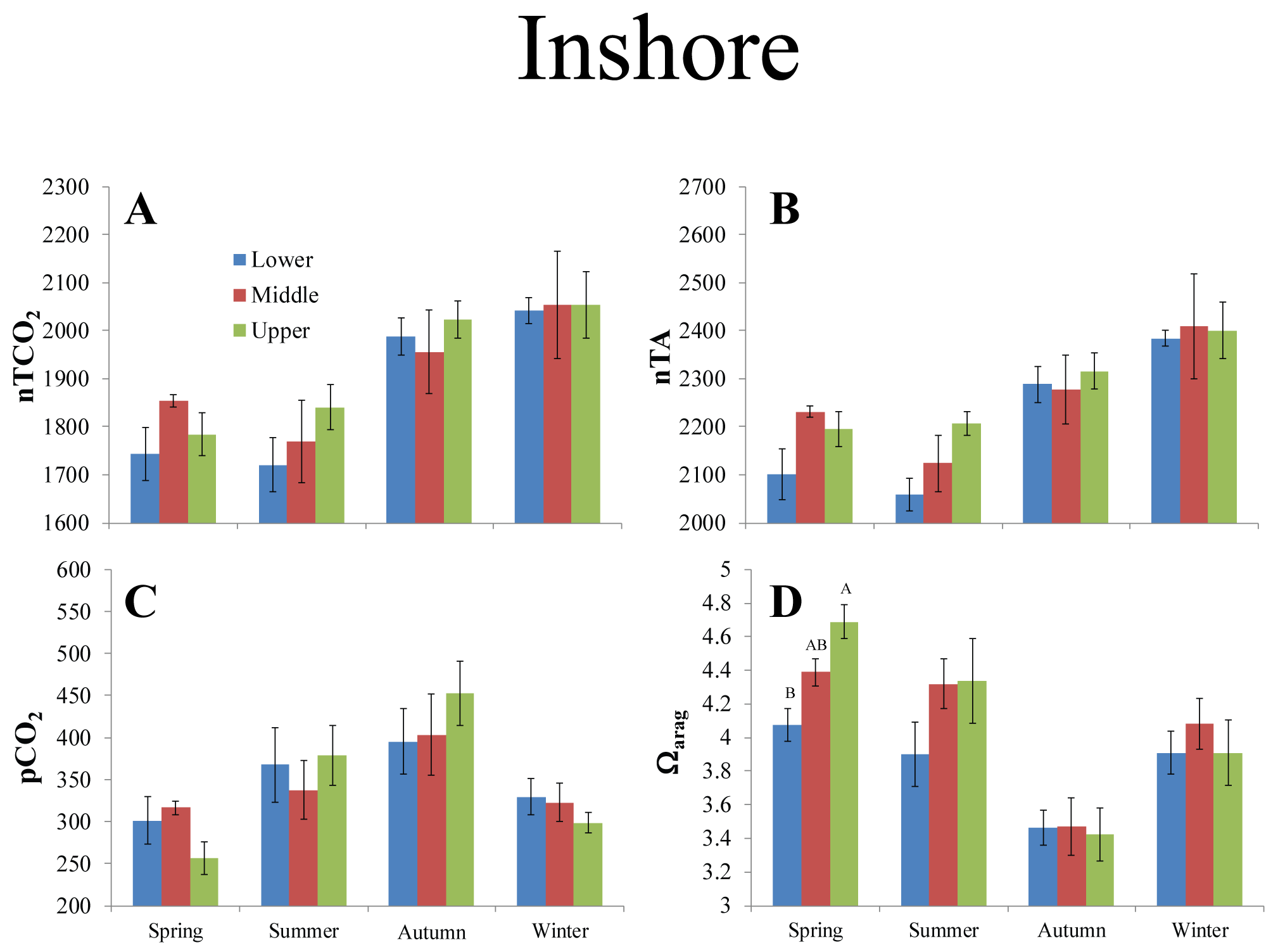

Supplement: Figure S1 — Carbonate Chemistry at Inshore Sites by Season. Mean values for (A) nTCO2, (B) nTA, (C) pCO2, and (D) Ωarag plotted by season for inshore sites from upper, middle, and lower keys. Error bars represent standard error of the mean. Means represent the average of mean values for each sampling excursion grouped by season. Non-matching letters indicate significant differences (t-tests, p<0.05). (TIF) [file pone.0041715.s001.tif]

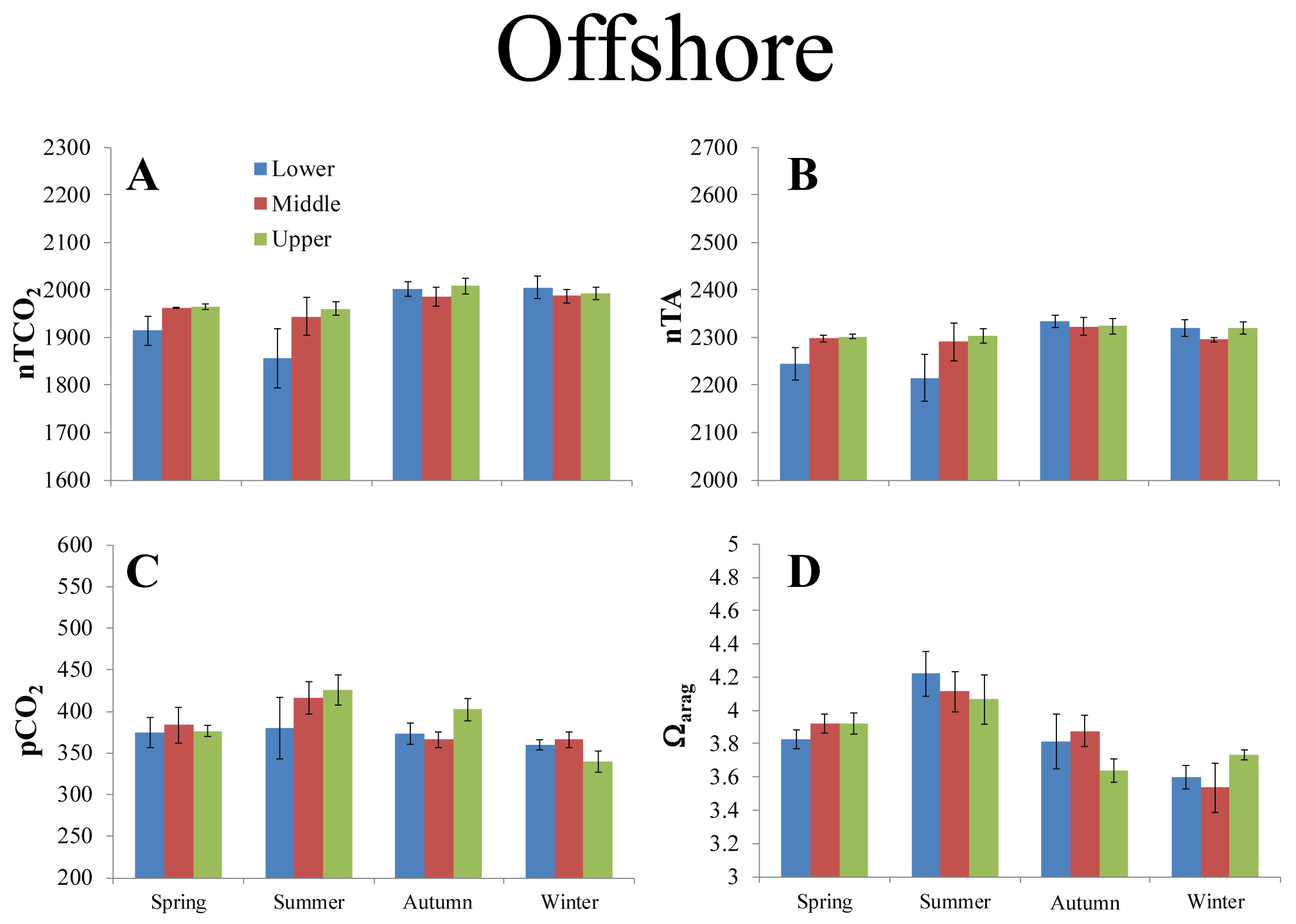

Supplement: Figure S2 — Carbonate Chemistry at Offshore Sites by Season. Mean values for (A) nTCO2, (B) nTA, (C) pCO2, and (D) Ωarag plotted by season for offshore sites from upper, middle, and lower keys. Error bars represent standard error of the mean. Means represent the average of mean values for each sampling excursion grouped by season. (TIF) [file pone.0041715.s002.tif]

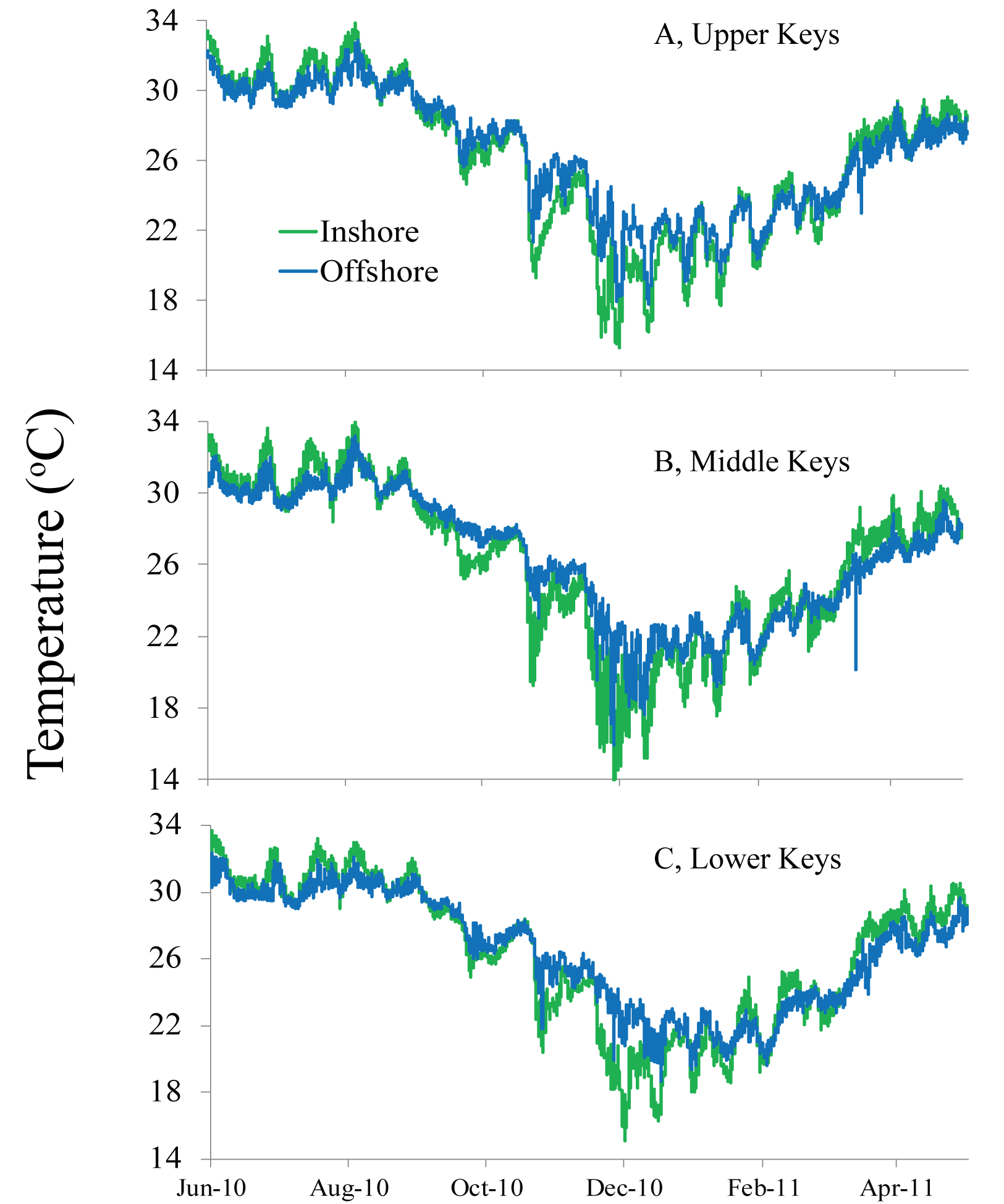

Supplement: Figure S3 — Seawater temperature at paired inshore and offshore sites. In situ temperature data, collected every 30 min from paired inshore (green lines) and offshore (blue lines) sites for (A) upper, (B) middle, and (C) lower Keys. Depths of temperature values are 5 m. (TIF) [file pone.0041715.s003.tif]
