# Supplementary material for: Ocean Acidification Refugia of the Florida Reef Tract
Source: PLoS One. 2012 Jul 27;7(7):e41715. doi: 10.1371/journal.pone.0041715 (PMC3407208; doi:10.1371/journal.pone.0041715)
Supplement: Table S1 — Timing of discrete sampling at paired inshore and offshore sites in the upper (UK), middle (MK), and lower (LK) Florida Keys. WS indicates sample taken aboard the R/V Walton Smith in coordination with the South Florida Program's repeat biophysical oceanographic cruises rather than small boat sampling (X). (DOC) [file pone.0041715.s004.doc]

| **Date** | **UK** | **MK** | **LK** |
| --- | --- | --- | --- |
| 14 April 2009 | X |  |  |
| 26-28 May 2009 | X |  |  |
| 28 Sep 2009 | X |  |  |
| 16-17 Feb 2010 | X |  |  |
| 8-9 Mar 2010 | WS | WS | WS |
| 24-25 May 2010 | X | X | X |
| 14-17 Jun 2010 | X | X | X |
| 29-30 Jun 2010 | WS | WS | WS |
| 23-24 Aug 2010 | X |  |  |
| 8-9 Dec 2010 | X | X | X |
| 21-22 Feb 2011 |  | WS | WS |
| 4-8 Apr 2011 | WS | WS | WS |
| 16-20 May 2011 | X | X | X |
| 7-8 Jun 2011 | WS | WS | WS |
| 20-22 Jun 2011 | X | X | X |
| 2-3 Aug 2011 | WS | WS | WS |
| 10-13 Oct 2011 | X | X | X |
| 20-21 Oct 2011 | WS | WS | WS |
| 11-12 Dec 2011 | WS | WS | WS |
| 27 Feb-2 Mar 2012 | WS | WS | WS |

**Table S1**
