# Supplementary material for: Ocean Acidification Refugia of the Florida Reef Tract
Source: PLoS One. 2012 Jul 27;7(7):e41715. doi: 10.1371/journal.pone.0041715 (PMC3407208; doi:10.1371/journal.pone.0041715)
Supplement: Table S2 — nTA-nTCO2 trendline equations and R2 by site and season. (DOC) [file pone.0041715.s005.doc]

| **Site** | **Season** | **Equation** | **R^2^** |
| --- | --- | --- | --- |
| **UK** | **Spring** | nTA = 0.7563 (nTCO_2_) + 33.718 | 0.57 |
|  | **Summer** | nTA = 0.551 (nTCO_2_) – 40.245 | 0.98 |
|  | **Autumn** | nTA = 0.3295 (nTCO_2_) – 7.3047 | 0.1 |
|  | **Winter** | nTA = 0.8596 (nTCO_2_) + 25.772 | 0.88 |
| **MK** | **Spring** | nTA = 0.5805 (nTCO_2_) – 4.199 | 0.997 |
|  | **Summer** | nTA = 0.6003 (nTCO_2_) – 62.337 | 0.962 |
|  | **Autumn** | nTA = 0.8932 (nTCO_2_) – 16.667 | 0.983 |
|  | **Winter** | nTA = 1.085 (nTCO_2_) + 42 | 0.98 |
| **LK** | **Spring** | nTA = 0.7436 (nTCO_2_) – 17.09 | 0.8794 |
|  | **Summer** | nTA = 0.9205 (nTCO_2_) – 30.502 | 0.8763 |
|  | **Autumn** | nTA = 1.0027 (nTCO_2_) – 40.908 | 0.8686 |
|  | **Winter** | nTA = 0.3159 (nTCO_2_) + 53.485 | 0.77 |

**Table S2**
