# Supplementary material for: Ocean Acidification Refugia of the Florida Reef Tract
Source: PLoS One. 2012 Jul 27;7(7):e41715. doi: 10.1371/journal.pone.0041715 (PMC3407208; doi:10.1371/journal.pone.0041715)
Supplement: Table S3 — Kruskal-Wallis results to identify seasonal differences within each of the six sites. (DOCX) [file pone.0041715.s006.docx]

**Table S3**

|  | **Inshore** |  |  | **Offshore** |  |  |
| --- | --- | --- | --- | --- | --- | --- |
|  | **Lower** | **Middle** | **Upper** | **Lower** | **Middle** | **Upper** |
| **TCO_2_** | p < 0.05 | p < 0.05 | p < 0.05 | ns | ns | ns |
| **nTCO_2_** | p < 0.05 | ns | p < 0.05 | p < 0.05 | ns | ns |
| **TA** | p < 0.05 | p < 0.05 | ns | ns | ns | ns |
| **nTA** | p < 0.05 | ns | p < 0.05 | p < 0.05 | ns | ns |
| **pCO_2_** | ns | ns | p < 0.05 | ns | ns | p < 0.05 |
| **Ω_arag_** | p < 0.05 | p < 0.05 | p < 0.05 | ns | ns | ns |
